# Short- & long-term effects of monetary and non-monetary incentives to cooperate in public good games: An experiment

**DOI:** 10.1371/journal.pone.0227360

**Published:** 2020-01-17

**Authors:** Mathieu Lefebvre, Anne Stenger

**Affiliations:** 1 BETA- University of Strasbourg, Strasbourg, France; 2 INRA and BETA- University of Strasbourg, Strasbourg, France; Middlesex University, UNITED KINGDOM

## Abstract

Using a common experimental framework, this paper addresses both the question of the short-term and the long-lasting effects of temporary monetary and non-monetary incentive mechanisms on increasing individual contributions to the public good. The results show that both punishments and rewards significantly increase contributions compared to the baseline, but that monetary sanctions lead to the highest contributions, whereas non-monetary sanctions lead to the lowest contributions. The four types of incentives display long-lasting effects, i.e., contributions do not go back to baseline levels directly after the withdrawal of the incentives. However, rewards appear to have much stronger persistent effects than sanctions, revealing some sort of delayed reciprocity.

## Introduction

An important question in public economics and environmental economics is how to promote contributions in social dilemma situations. In many contexts, individuals face a trade-off between self-interest and group interest, and free-riding is a pervasive phenomenon of social life. Well-known examples of such situations include biodiversity conservation, depletion of a common resource pool, charitable giving or private provision of a public good. The introduction of incentive programs can increase the overall welfare by increasing individual contributions, but such schemes are costly to implement and are often temporary for political and/or budgetary reasons. Thus, there is an increasing interest in identifying the long-term effect of short-term public policies as well as identifying which types of incentives work the best to induce a change in individual behavior. It has been shown that it is important to go beyond the study of short-term effects in order to understand the effectiveness of incentives and, more globally, the implications for policy design [1]. Whereas many incentive programs reveal short-term effects, evidence concerning long-term effects is often much more limited. When we look at how individual decisions and behaviors evolve over a given horizon, we are led to question the existence and links between incentives, the type of incentives and their dynamics over time.

In this paper, we compare the effectiveness of various incentive mechanisms to achieve and sustain high levels of cooperation in a voluntary contribution public good game and how they differ in terms of inducing long-lasting cooperation when they are only provided for a temporary period. The originality of our paper is twofold. First, in a repeated public good in a fixed-partner design, we compare treatments in which monetary and non-monetary incentives are available. We also compare positive (rewards) and negative (punishments) incentives. While the effect of monetary sanctions and rewards is well documented, their comparative effectiveness with non-monetary incentives in a common framework is less well-established, especially when the social dilemma takes the form of a contribution to a public good. The second originality of our paper is that we look at the long-lasting effects of each one of those incentive schemes when they are removed after a fixed number of periods.

Previous experiments have shown that introducing monetary incentives such as formal sanctioning or rewards increases contributions to a public good and slows down the decay observed with repetitions [2–3]. Interestingly, it has also been shown that non-monetary incentives can sustain cooperation as well [4–5]. In particular, in a design similar to that of [2], [4] introduced non-monetary punishments such as expressions of disapproval. They showed that both monetary and non-monetary sanctions initially increase contributions. [5] and [6] compared the effectiveness of sanctions and rewards and found that both increase individual contributions, whereas sanctioning appears to be a more effective mechanism for sustaining high levels of cooperation. There is also an increasing literature that has shown the effectiveness of persuasive messages, social interactions and nudges on voluntary contributions. Since the subject’s attitude toward contribution is affected by social interaction through subjective norms, behaviors may be modified by introducing normative statements that act on intentions [7]. Recently, [8] and [9] showed that introducing moral messages has a positive and significant effect on contributions. However, to our knowledge, no study has yet attempted to assess the relative effectiveness of both monetary and non-monetary sanctions and rewards within a comparable framework.

Furthermore, understanding the extent to which a short-term policy has persistent effects also remains an open question. The theoretical literature largely ignores the long-lasting effects of interventions that aim at changing behaviors. If we assume that individuals rationally choose among their opportunities according to their preferences, then individuals’ opportunities can be shaped by providing incentives for desired behavior and their preferences shaped by increasing their taste for desired behavior [10]. Identifying which channel is the driver of change, if any, is a difficult task, and there are several reasons why an incentive program could have long-lasting effects such as the updating of information [11–12], the adoption of new habits [13], new social norms [14], etc. Recent empirical papers have focused on the persistence of the impact of a policy once it is suspended, and the results are mixed and depend on the data, the framework or the context and the estimation method. In their paper [15] demonstrate in an experimental design that policy interventions such as push measures (rebates and minimum dotation rules) are more effective than nudges (default, social information). Their effects are persistent over time, but only when the context remains the same over time (dictator game) without significant spillovers in a different subsequent game like the prisoner’s dilemma.

Studying the effect of energy conservation programs in the US and in Brazil, [1] and [16], respectively, showed that even if the initial effect is reduced once the program is stopped, temporary policies tend to lead to a long-lasting reduction in electricity use. [17] and [18] found that economic incentives can induce habit formation for exercising at a gym. On the contrary, many concrete cases from Payments for Ecosystem Services programs reveal the lack of persistence at the end of the agreement [19–20]. Yet evidence is not limited to monetary incentives and it has been shown that the content and timing of given information by behavioral energy conservation programs can impact the short- and long-run behaviors [1]. In particular, [21] found that the effect of moral suasion on energy-saving quickly diminished after repeated interventions. In a recent paper on eco-driving, [22] demonstrated some significant effects of non-monetary reward on the individual reduction of fuel consumption, but only temporarily since it attenuates over time without backsliding. Looking at the effect of different norm-based strategies on the long-run patterns of residential water use, [23] found that norm-based messages influence water demand but that the effectiveness of such messages wanes over time.

Few experimental studies have attempted to assess the long-lasting effect of temporary incentives and, to our knowledge, no study has yet to compare the long-lasting effects of monetary and non-monetary incentives. In a minimum-effort game (teamwork framework), [24] and [25] explore the effect of the introduction of incentives once the groups have converged to an inefficient equilibrium and the effect of a subsequent removal of the incentives. While both papers show the effectiveness of the incentives in improving coordination, [25] found few persistent long-term effects with the effort going back to its pre-incentive level. On the contrary, [24] found that reductions in the incentives have little effect on later behavior. Closer to our paper, [26] used a repeated linear public good game to investigate whether providing strong cooperation incentives for only a number of periods spills over to later periods. Their results are similar to [25], i.e., cooperation rapidly deteriorates once monetary incentives are reduced. Recently, [27] also showed that past outcomes can shape tax compliance in the future once a major institutional change occurs. They point to the quality of tax institutions in the past as a good predictor of future behavior.

Anticipating our results, we show that monetary and non-monetary punishments and rewards significantly increase contributions compared to the baseline. Monetary sanctions and non-monetary rewards are the most effective in increasing cooperation. The four incentive schemes also reveal long-lasting effects on contributing behaviors, but sanctions and rewards act differently in driving future contributions. Rewards, in particular, appear to have much stronger persistent effects than sanctions. This could be due to some kind of delayed reciprocity since those who have been highly rewarded contribute more once the rewards have been removed. These effects are not impacted by how long the subjects have been incentivized.

The following section describes the experimental design as well as the predictions and procedures. Section 3 presents the results and the last section provides a conclusion.

## Materials and methods

### Experimental design

Our experimental setup consists of a repeated Voluntary Contribution Mechanism (VCM) played by fixed groups of four subjects for 30 periods [28]. At the start of each period, subjects receive an endowment of 20 tokens each and have to decide, simultaneously and without the possibility of communicating with the other group members, how many tokens they want to keep for themselves and how many tokens they want to invest in a project. Each investment made in the project yields a payoff of 0.4 tokens to each of the four members of the group. Therefore, the earnings of individual *i* who contributes *c_i_* to the project in a given period are expressed as:

$$\pi_{i}^{B}=20-c_{i}+0.4\sum_{k=1}^{4}c_{k}$$

Table 1 provides the basic design information, and instructions for *MP* are presented in the Appendix. We considered four treatment conditions in addition to the *Baseline* described above: *Monetary Punishment (MP)*, *Non-monetary Punishment (NMP)*, *Monetary Reward (MR)* and *Non-monetary Reward (NMR)*. In the four supplementary treatments, each subject participated in two sequences of 15 decision periods.

**Table 1:** Treatment conditions.

	Subjects	Sequence I (Periods 1–15)	Sequence II (Periods 16–30)
*Baseline*	40	VCM	VCM
*Monetary Punishment (MP)*	40	VCM + Punishment	VCM
*Non-monetary Punishment (NMP)*	40	VCM + Punishment	VCM
*Monetary Reward (MR)*	40	VCM + Reward	VCM
*Non-monetary Reward (NMR)*	40	VCM + Reward	VCM

In Periods 1–15, each period consisted of a two-stage game. In Stage 1, subjects play a standard VCM in which they have to decide, simultaneously and without the possibility of communicating with the other group members, how to allocate their 20-token endowment.

At the beginning of the second stage, subjects are informed of the contribution levels of each of the other members of their group. One alternative would have been to present each member’s individual income. However, [29] has shown that giving the individual income instead of the individual contributions reduces the effectiveness of the punishment mechanism. Individual decisions are not linked to subject identifiers and contributions are presented in ascending order in each period so that subject-specific reputations cannot develop across periods. Depending on the treatment condition, subjects can make a second decision in Stage 2:

- In the *Monetary Punishment (MP)* treatment, subjects could assign zero to ten punishment points to each of the three other group members. Each point, *p_ij_*, assigned by subject *i* to subject *j* lowered subject *j*’s income by one token. There was also a cost of 0.25 tokens for subject *i* associated with each point allocated. The effectiveness of the punishment mechanism has been shown to be related to the mix of cost-impact of the punishment. [30] showed that a low cost-high impact punishment is the most effective mechanism. We opted for a 1 to 4 ratio. This implies that payoffs at the end of Stage 2 and, thus, for the given period, are expressed as:
  $$\Pi_{i}=\pi_{i}-\sum_{j\neq i}p_{ji}-0.25\sum_{j\neq i}p_{ij}$$
  The choice of punishment points is restricted to the actual earnings from the first stage, but the earnings at the end of a period can be negative depending on the number of punishment points distributed and received.
- In the *Non-monetary Punishment (NMP)* treatment, the rules were similar to those of *MP*, except that each point awarded to the subjects had no effect on their final earnings and was costless to assign. As in *MP*, each subject had the opportunity to assign between 0 and 10 points to each member of the group. In a similar framework to [4] these points corresponded to the level of disapproval of the subject’s contributions in the first stage. Ten points corresponded to the highest level of disapproval and zero points to the lowest level of disapproval.
- In the *Monetary Reward (MR)* treatment, the mechanism was identical to the *MP* treatment, except that instead of assigning points to sanction other group members, subjects could use points to reward them. Subjects could assign zero to ten reward points. Each point, *p_ij_*, assigned by subject *i* to subject *j* increased subject *j*’s income by one token. As in *MP*, there was a cost of 0.25 tokens for the subject assigning the points associated with each point allocated. Thus, rewards constituted a pure redistribution of earnings. This implies that payoffs at the end of Stage 2 are expressed as:
  $$\Pi_{i}=\pi_{i}+\sum_{j\neq i}p_{ji}-0.25\sum_{j\neq i}p_{ij}$$
- In the *Non-monetary Reward (NMR)* treatment, the rules were similar to those of *MR*, except that, as in *NMP*, each point awarded to the subjects had no effect on their final earnings and was costless to assign. The only opportunity for subjects to express their approval of the group members’ contributions was by assigning 0 to 10 reward points.

In each of these four treatments, after having assigned points (either sanctions or rewards), subjects were informed of their earnings, including any punishment (reward) they imposed or received. Subjects were also informed of the total number of punishment (reward) points they received, but could not identify which of the other subjects imposed the punishment (rewards). Furthermore, subjects were not informed of the number of punishment (reward) points that the other group members received.

In Periods 16–30 of the four incentivized treatments (*MP*, *MR*, *NMP* and *NMR*), each period is identical except that there is no Stage 2, i.e., no more opportunities for rewards or sanctions. Each period consists of a standard VCM as in the *Baseline*. This was clearly stated in the instructions from the very beginning of the experiment and in all treatment conditions. Subjects are also aware that they play a finitely repeated game with a final period.

### Predictions

It is assumed that subjects care only about their monetary payoffs, are fully rational and that it is common knowledge that they should not contribute in the *Baseline* and that they should also abstain from costly punishment or reward [31–32]. However, we know that we can expect positive contributions in the *Baseline* followed by a continuous decay until the last period due to the presence of conditional cooperators [33]. As shown by [32], if we assume that subjects display social preferences in the manner of [34] where they compare their own payoff with the payoff of each member in their group, there is a multiplicity of equilibrium. Let the utility of a subject *i* in a group of four subjects that depends on the set of monetary payoffs in the group *x =* (*x1*, *x2*, *x3*, *x4*) be expressed in the following form:

$$U_{i}(x)=x_{i}-\alpha_{i}\frac{1}{4-1}\sum_{j\neq i}max\{x_{j}-x_{i},0\}-\beta_{i}\frac{1}{4-1}\sum_{j\neq i}max\{x_{i}-x_{j},0\}$$

The second term on the right-hand side of the equation captures the utility losses from disadvantageous inequality, and the third term the losses from advantageous inequality. Where *β_i_*≤*α_i_* and 0≤*β_i_*<1. [32] showed that if at least one member cares relatively little about advantageous inequality (for example, *β_i_*<0.6), the only equilibrium is complete free-riding and no subject contributes to the public good. Otherwise, there is a multiplicity of equilibriums with all members contributing the same amount that can take any value equal to or greater than zero.

This unstable cooperation has been shown to be fixed by the introduction of sanctions or rewards [2–3–4–32]. This means that it can be expected that there are cooperators that are willing to engage in the punishment of free riders as well as in the rewarding of good contributors. However, here as well, the effectiveness of the punishment or the reward mechanism will depend on the presence of subjects who care little about advantageous inequality. Given the previous results, punishments should lead to higher contributions than rewards, and monetary incentives should lead to higher contributions than non-monetary ones. Whether or not we observe positive contributions during period 1–15, predictions for periods 16–30 are not clear-cut. As pointed out by [26], after the removal of incentives, predictions about contribution levels depend on the hypothesis retained. If we assume that the incentives primarily influence contributing behavior, contribution levels should go down to the *Baseline*, like in [25]. If we assume that the incentives improve coordination and possibly create trust and self-image that should influence later interactions, we should not observe much change from what we obtain in periods 1–15 [24]. [35] insist on the image concern as a reason to maintain high average contributions even when strong material incentives have been removed. Finally, it might be that the contribution levels decrease to a level below the *Baseline*. However, this would generally happen with monetary incentives that have been shown to backfire in some cases [24–36–37]. This means that incentives can have different long-lasting effects depending on their intrinsic nature. Greater persistent effects with rewards and with non-monetary incentives can be expected if they impact self-image more than punishments and monetary incentives. Although the effect of all these incentives has been shown to be strong, their long-lasting effect is somewhat unknown.

### Procedures

A total of 200 subjects participated in ten sessions. All of the subjects were recruited from a list of experimental subjects maintained at BETA, University of Strasbourg, France, using ORSEE software [38]. The experiment was conducted in French. The English translation of the experiment instructions is available in the Appendix. The original French instructions are available upon request. Subjects sign an informed consent agreement when entering the database. They then sign up for experiments on a voluntary basis and are randomly allocated to sessions. Subjects were on average 20.5 years of age, and 49% of the subjects were female. They were involved in a wide range of fields but 26.5% of them were studying economics or business management.

The experiment was computerized. Upon arrival, each subject was randomly assigned a computer. The instructions were read aloud by the experimenter and, before starting, a comprehension questionnaire was administered to check that the rules were well understood. All questions were answered in private. Once the 30 periods were completed, the screens displayed the total cumulative gains for the experiment and the subjects answered a post-experiment questionnaire. Then, at the end of the session, subjects were paid their earnings in a separate room and in private. The conversion rate was 30 tokens to €1. Average earnings were €25.8 (standard deviation = 4.1). The experiment lasted an average of 80 minutes.

## Results

In order to assess the difference between the four incentive schemes both in terms of their effectiveness and their long-lasting effects, we present an average of the contributions to the public good and for each period. An analysis of the choices of punishing or rewarding are not presented but are available upon request.

### *Baseline* vs. treatment conditions

Table 2 presents the average contributions in each treatment by comparing the initial sequence of 15 periods with the last 15 periods. In each sequence, a test of significant difference with the *Baseline* is performed. Unless specifically noted, we report the significance levels of a two-sided Mann-Whitney rank-sum test taking group averages as the unit of observation. Table 2 shows that in general, the individual contributions are significantly much higher in *MP*, *NMP*, *MR* and *NMR* than in the *Baseline* for periods 1–15. This is also true when we look at the subsets of the periods, except for *MR* that is not significantly different from the *Baseline* during the first five periods. In the first sequence of the game, monetary sanctions and non-monetary rewards lead to higher contributions than both non-monetary sanctions (p<0.001 and p = 0.025, respectively) and monetary rewards (p<0.001 for both). The difference between *MP* and *NMP* is significant during periods 1–5 (p = 0.02), periods 6–10 (p = 0.018) and periods 11–15 (p = 0.008). The difference between *MP* and *MR* is significant during periods 1–5 (p<0.001), periods 6–10 (p = 0.098) but not during periods 11–15 (p = 0.198). The difference between *NMR* and *NMP* is significant during periods 1–5 (p = 0.002), but not significant during periods 6–10 (p = 0.121) and significant during periods 11–15 (p<0.001). The difference between *NMR* and *MR* is significant during periods 1–5 (p<0.001), periods 6–10 (p = 0.055) but not during periods 11–15 (p = 0.483). The effectiveness of non-monetary rewards is noticeable since the average contributions are almost equal to those in *MP*, which contradicts recent evidence about the lack of effectiveness of non-monetary rewards [5]. However, the study by [5] considered a minimum-effort game and not a VCM.

**Table 2:** Mean contributions.

	Sequence 1				Sequence 2			
Periods	1–15	1–5	6–10	11–15	16–30	16–20	21–25	26–30
*Baseline*	7.3 (6.6)	9.9 (6.9)	6.8 (6.3)	5.1 (5.8)	3.0 (4.5)	3.8 (5.0)	3.2 (4.3)	2.0 (3.8)
*Monetary Punishment (MP)*	15.1*** (7.6)	15.9*** (6.6)	15.1*** (7.6)	14.3*** (8.4)	8.9*** (8.9)	10.7*** (8.7)	8.6*** (9.0)	7.4*** (8.6)
*Non-monetary Punishment (NMP)*	12.9*** (7.5)	14.4*** (6.6)	13.0*** (7.9)	11.4*** (7.6)	5.1*** (6.5)	7.5*** (7.4)	4.4 (5.9)	3.5 (5.4)
*Monetary Reward (MR)*	12.8*** (7.7)	12.0 (7.3)	12.6*** (8.0)	13.6*** (7.7)	7.5*** (8.1)	9.8*** (8.3)	8.0*** (8.0)	4.7 (7.2)
*Non-monetary Reward (NMR)*	15.1*** (5.5)	15.7*** (4.7)	14.9*** (5.6)	14.6*** (6.2)	8.2*** (7.6)	11.2*** (7.5)	8.4*** (7.4)	5.0*** (6.4)

Standard errors are in parentheses.

***, **, and * stand for significance differences at the 1%, 5% and 10% level, respectively, according to a two-sided Mann-Whitney test of difference with the *Baseline*, taking the group average as a unit of observation.

**Fig 1:**
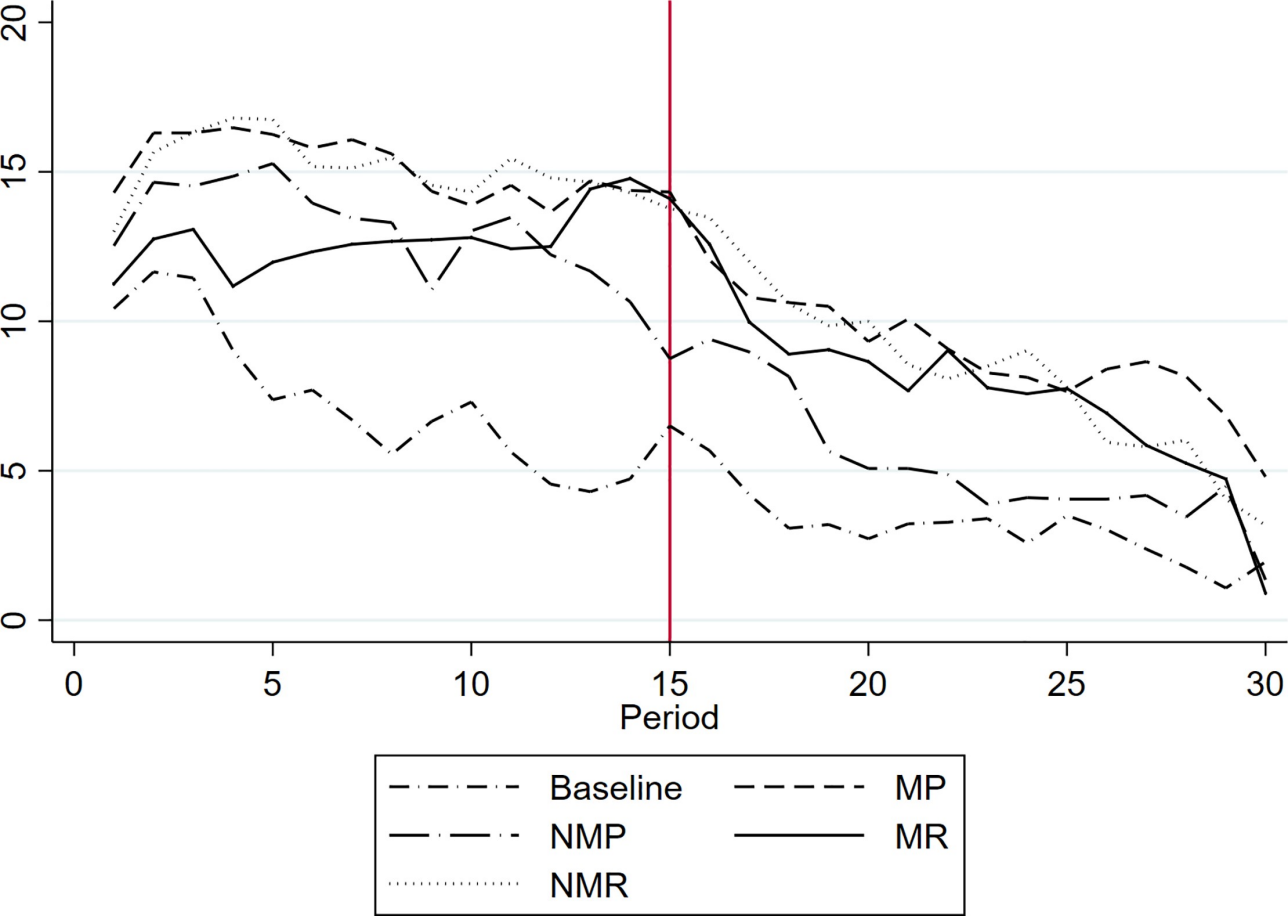
Average individual contributions.

Fig 1 shows the time series of individual contributions per period in the *Punishment* and the *Reward* treatments compared to the *Baseline*. Contributions at the group levels display similar results and are available upon request. The bold line indicates the *Baseline* contribution. The pattern of contribution in the *Baseline* is consistent with that observed in previous studies [28–33]. Contributions start from about 50% of the endowment and then continuously decrease until period 30. During the first 15 periods, the contributions in the four incentivized treatments are well above the *Baseline* and appear to be more stable than in the *Baseline*, which is also in line with previous studies (see, i.e., [39]).

*Result 1*: *(a) Both monetary and non-monetary punishments and rewards significantly increase contributions compared to the baseline, but (b) monetary sanctions and non-monetary rewards are more effective and lead to the highest level of contributions*.

From period 16 onwards, opportunities to punish or to reward are removed from every treatment. Table 2 shows that during periods 16 to 30, the contributions are still significantly higher, on average, than the *Baseline* for all treatments. Interestingly, the contributions in the *NMP* treatment are much lower than in the three other incentivized treatments, and when we look at the subsets of the periods, they are only significantly different from the *Baseline* for the first five periods of Sequence 2. In Fig 1, we observe that in period 16, contributions in the four incentivized treatments do not immediately drop to the *Baseline* level. In *MP* and *MR*, we observe a decrease of about 25% after the end of the opportunity to punish or reward but still higher than the *Baseline*. We do not observe such a drop in the *non-monetary* treatments, *NMP* and *NMR*. The positive difference with the *Baseline* tends to decrease faster for sanctions than for rewards. However, except for the last periods, contributions remain at a high level for several periods after the incentive has been withdrawn. Table 2 shows that the difference with the *Baseline* is significant until period 20 for non-monetary sanctions and until period 25 for monetary rewards. There is evidence of a “last period effect” [40–41]. A striking result in our repeated experimental setup is that non-monetary rewards perform well in producing long-lasting contributions.

The differences between treatments are confirmed by the regression results in Table 3. The first two columns present Tobit estimations for the individual contributions during periods 1–30 and 1–15. The specification includes control for age, gender and if the subject is a student in economics or management. In addition to treatment variables, we also introduce a period variable as well as the relative contribution to the group in the preceding period. The reference is the *Baseline* treatment. Generally speaking, the results confirm the strong effect of our four incentivized treatments on the individual contribution. *Monetary sanctions* have the strongest effect, followed by *Non-monetary rewards*. *Non-monetary punishments* have a smaller but significant effect on contributions. Those who were positively far from the group contribution in the preceding period contribute more. We can observe a decline in the level of contribution over time. The third column presents the same estimation as in specification (1) and (2) but for the periods 16–30. The results confirm previous findings. Although punishments and rewards can no longer be applied, we still observe significant deviations from the *Baseline* treatment. *Monetary sanctions* have the biggest impact once those incentives are no longer present. *Non-monetary punishment* has a small and marginally significant impact.

**Table 3:** Determinants of individual contributions, Random-effects Tobit models.

	(1)	(2)	(3)	(4)
	Periods 1–30	Periods 1–15	Periods 16–30	Periods 16–30
*MP*	13.854***	23.977***	6.598***	25.700***
	(1.026)	(1.243)	(0.849)	(3.898)
*MR*	11.399***	13.359***	5.175***	25.183***
	(0.763)	(0.845)	(0.819)	(3.442)
*NMP*	7.350***	9.196***	1.609*	15.249***
	(0.768)	(0.728)	(0.852)	(3.358)
*NMR*	15.536***	9.258***	5.629***	15.637***
	(0.812)	(1.887)	(0.838)	(3.378)
Relative contribution in t-1	0.128***	0.095***	0.036*	-0.000
	(0.024)	(0.032)	(0.020)	(0.034)
Period	-0.765***	-0.372***	-0.486***	-0.488***
	(0.018)	(0.044)	(0.024)	(0.099)
MP * Periods				-0.502***
				(0.148)
MR * Periods				-0.663***
				(0.141)
NMP * Periods				-0.313**
				(0.137)
NMR * Periods				-0.582***
				(0.136)
Constant	17.299***	8.739**	14.582***	19.836***
	(2.605)	(3.818)	(2.622)	(4.399)
*N*	5800	2800	2800	2800
*Log-likelihood*	-11871.82	-5676.47	-8068.08	-5486.57

Standard errors are in parentheses. All regressions contain a control for age and a dummy for gender as well as a dummy if the subject is studying economics or management.

* *p* < 0.1

** *p* < 0.05

*** *p* < 0.01.

In order to identify diverging behavior among treatments and over time, specification (4) introduces interaction effects between treatment variables and time. Results show that after the first 15 periods, subjects in each of the four treatments with incentives contribute more than in the *Baseline*, but the interaction of treatment variables with time show that the contributions decrease more rapidly than in the *Baseline*. This is not surprising since we observe a kind of convergence at the end of the 30 periods between all treatments, revealing an effect of a known end of game.

*Result 2*: *(a) MP, NMP, MR and NMR have long-lasting effects since contributions do not go back to baseline levels directly after the withdrawal of the incentives but (b) monetary punishments appear to have stronger persistent effects than other incentives. (c) Non-monetary and monetary rewards have the same effectiveness on contributions*

### Periods 1–15 vs. periods 16–30

In order to identify a potential restart effect, we compare the contributions in the *Baseline* for periods 1 to 15 with the contributions in the four treatment conditions for periods 16 to 30. We do not actually observe significant differences except for the NMP treatment (p<0.05) for which Table 2 displays lower contributions than in the *Baseline*. Refining this analysis by comparing contributions during periods 1–5 in the *Baseline* with contributions during periods 16–20 in our treatments conditions, we find no significant differences between the *Baseline* and MP (p = 0.512), MR (p = 0.716) and NMR (p = 0.318). The only significant difference is observed for NMP (p = 0.048).

**Fig 2:**
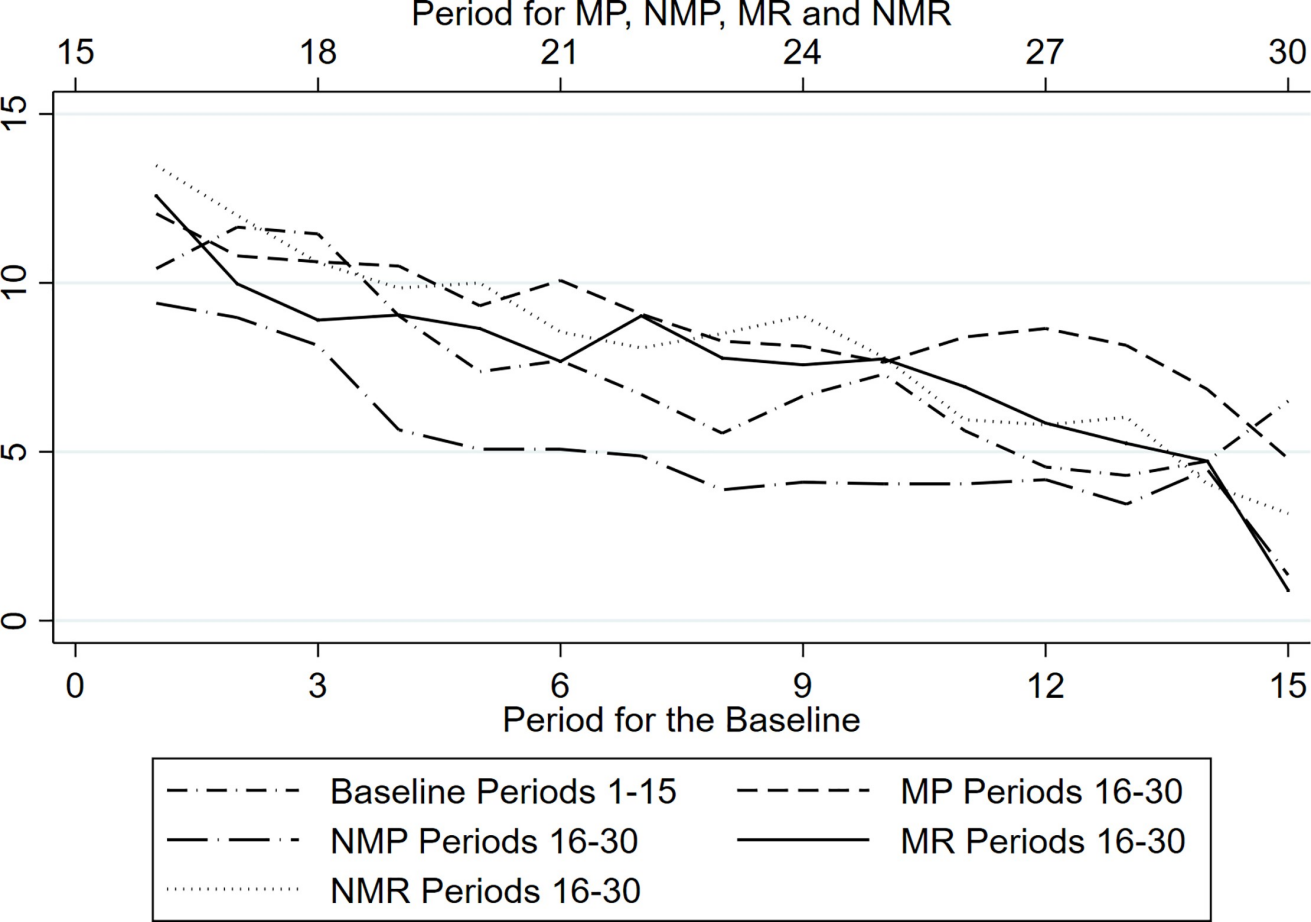
Average contributions before period 16 for the *Baseline* and after period 15 for *MP*, *NMP*, *MR* and *NMR*.

Fig 2 clearly confirms this result. In this figure, the contributions in the *Baseline* during the first 15 periods (lower x-axis) are compared to the contributions in the treatment conditions when incentives are removed (upper x-axis). We hardly observe differences between contributions in the treatments. Fig 2 shows that as of period 16, the removal of the incentives could act as a restart effect since the contributions in period 16 are on the same level as in the *Baseline* in period 1. These comparisons tend to mitigate the long-lasting effects we previously identified. However, our design does not provide a proper stoppage step that would allow us to properly identify a true restart effect [42].

### The dynamics of incentives

**Fig 3:**
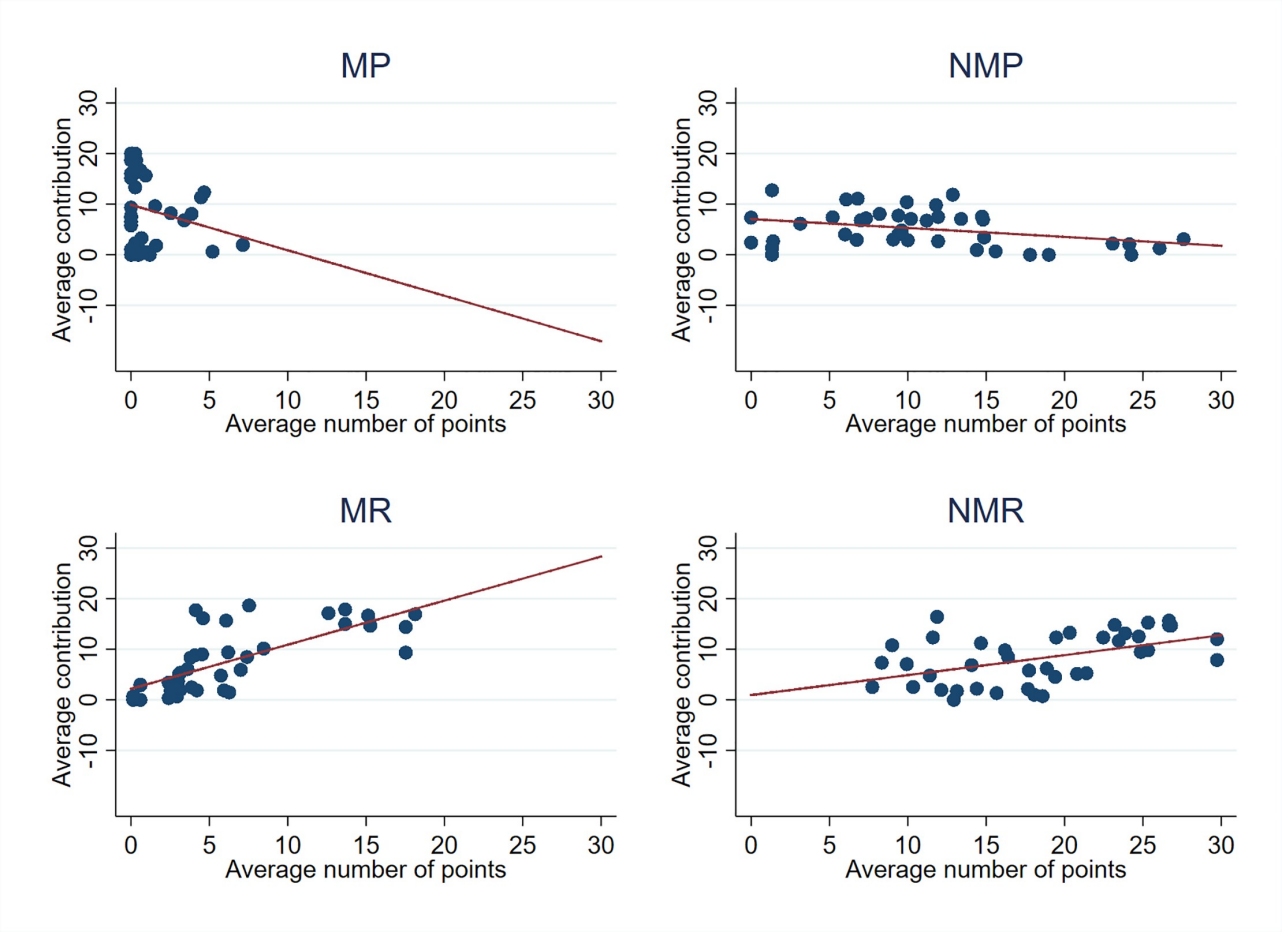
Average contributions in periods 16–30 according to the average number of points received in periods 1–15.

In order to gain a better understanding of the dynamics of individual contributions, we investigated how the individual decisions to contribute in the last periods are influenced by the incentives provided in the first periods. Fig 3 presents the average contribution in period 16–30 according to the number of points received during the periods 1–15. The effect of receiving monetary or non-monetary points on the later contributions is different according to the treatment, which suggests that rewards and sanctions can have a different meaning in the long run. In general, those who have been punished a lot tend to contribute less afterwards, whereas those who have been awarded a lot contribute more. In the punishment treatments, a part of those who did not contribute much in the first part of the game (and are then likely to be sanctioned) are likely to maintain their initial behavior throughout the experiment. They may even contribute less in the last periods to gain back what they have lost because of punishment points in *MP*. On the contrary, in the rewards treatments, we observe some sort of delayed reciprocity behavior since those who have been highly rewarded are those who contribute more once reward opportunities have been removed.

*Result 3*: *(a) In MP, those who contributed less during the first periods and have been highly sanctioned are also those who contribute less once the sanction opportunity has been removed. (b) In MR and NMR, we observe some kind of delayed reciprocity since those who have been highly rewarded are those who contribute more once the rewards have been removed*.

Table 4 confirms these results by presenting regressions by treatment for periods 16–30 when we introduce the total number of points received during periods 1–15 and the average contribution during this first sequence as explanatory variables as well as their interaction to see whether punishments and rewards affect low and high contributors differently. We also tried using the average number of points received and found that it does not change the conclusions either. Results are available upon request. Surprisingly, monetary and non-monetary sanctions have different effects on contributions in the last periods. While receiving monetary punishments appears to increase low contributing subjects’ contributions for the rest of the game, the non-monetary sanction has an opposite effect. Table 4 also shows that high contributors that were not monetarily sanctioned tend to contribute more afterwards, which is not the case with non-monetary sanctions. Finally, we observe that those who contributed more but were still sanctioned also contribute more in both treatments. Monetary punishments somehow discipline low contributors (free riders) in the future, which is not the case of the non-monetary sanction.

**Table 4:** Determinants of contributions per treatment in periods 16–30: Random-effect Tobit model.

	(1)	(2)	(3)	(4)
	*MP*	*NMP*	*MR*	*NMR*
N points received	0.117*	-0.101***	-0.107	-0.067*
	(0.052)	(0.021)	(0.099)	(0.039)
Av. Contrib. periods 1–15	3.075***	-0.832***	1.502***	1.018
	(0.290)	(0.310)	(0.282)	(1.028)
N points received * Av. Contrib.	-0.024***	0.009***	0.006**	0.015**
	(0.005)	(0.002)	(0.002)	(0.003)
Period	-1.177***	-0.730***	-1.112***	-1.003***
	(0.169)	(0.106)	(0.116)	(0.099)
Constant	-20.080*	44.068***	21.686*	0.674
	(11.225)	(10.266)	(11.498)	(13.674)
*N*	560	560	560	560
*Log-likelihood*	-828.73	-1159.72	-1057.61	-1289.11

All regressions contain a control for the periods and a dummy for gender as well as a dummy if the subject is studying economics or management. Standard errors are in parentheses.

* *p* < 0.1

** *p* < 0.05

*** *p* < 0.01.

We do not observe the same diverging effect with rewards. Both monetary and non-monetary reward points reveal the same effects. Low contributors who received a large number of reward points, either monetary or non-monetary, contribute less during the last 15 periods than those who received fewer points, but this is only marginally significant for *NMR* and not significant for *MR*. There are very few of these subjects in any case. However, the effect is positive and significant at 5% for the high contributors of the first sequence. These results could explain why we observe a rather long-lasting impact of the reward treatments.

### Varying the length of the incentive periods

**Fig 4:**
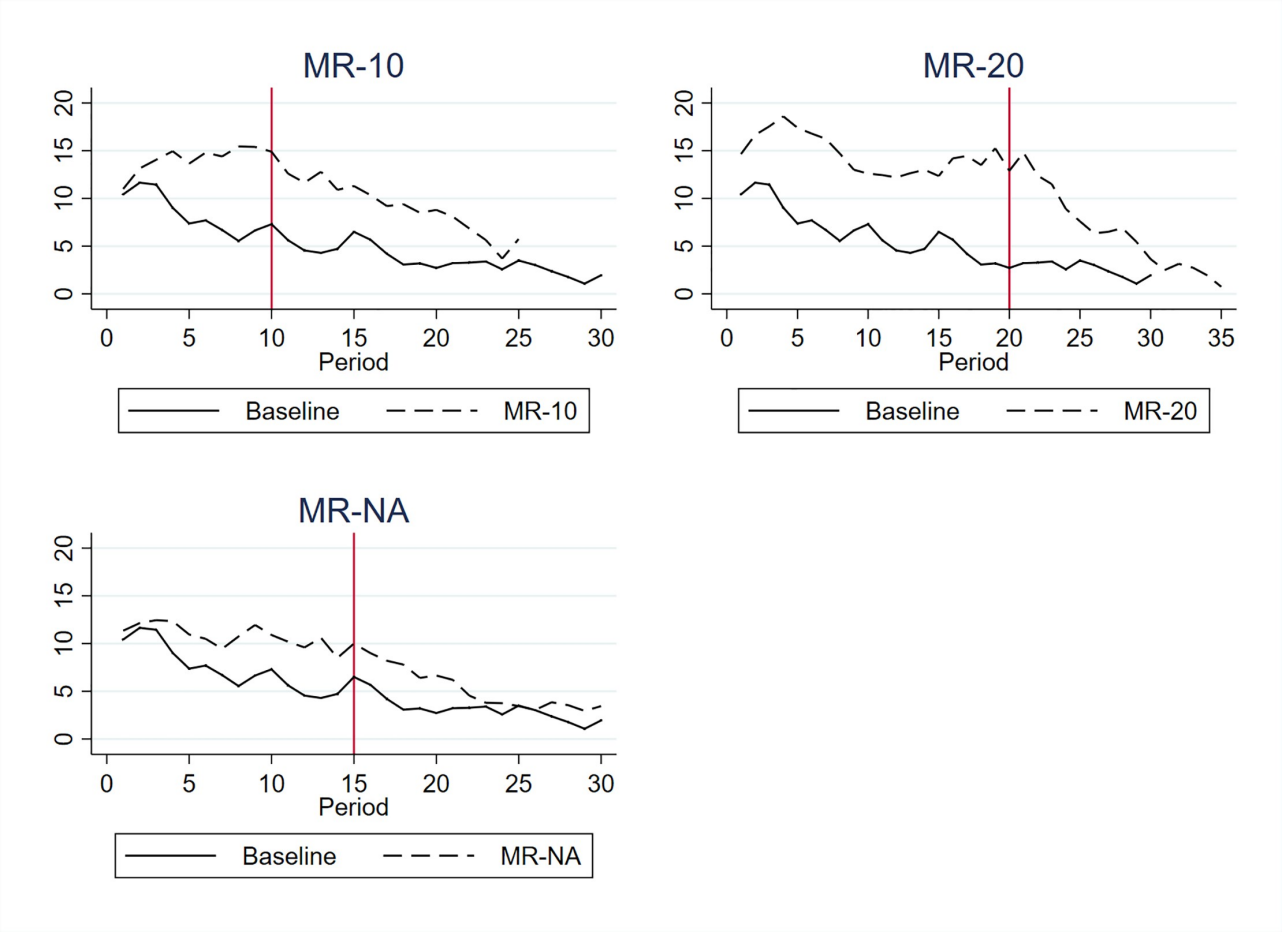
Average contribution–Monetary rewards with varying lengths of incentivized and un-incentivized sequences.

As a robustness test, we also ran three additional treatments in which we looked at monetary rewards when the length of the incentivized period varies or when it is uncertain. We only looked at rewards since they turned out to be the most effective in ensuring long-lasting effects. Since monetary and non-monetary rewards displayed similar effects, we only ran additional treatments with the monetary incentive. In two treatments, in which 20 subjects participated, we varied the number of periods during which rewarding is available: either 10 periods (MR-10) or 20 periods (MR-20). In both treatments, once the incentives are removed, the subjects play 15 periods of a regular VCM. A third additional treatment, called MR-NA (also with 20 subjects), makes the end of the second sequence where no incentives are available uncertain. Once the first 15 periods elapsed, we did not tell the subjects how many periods were left until the end of the experiment. Fig 4 shows the average contribution per period. Interestingly, when the length of the incentivized sequence is shortened or increased, we still observed the effectiveness of the rewards and a long-lasting effect once the incentive is removed. In both case the contributions are significantly higher than in the *Baseline*. In the case of an uncertain end of the game, we have mixed results. The possibility of rewards induces significantly higher contributions than in the *Baseline*, although the effectiveness is lower than in other rewarding treatments. Once the possibility of incentive is removed, we still observe higher contributions, but the difference with the *Baseline* is no longer significant. The uncertainty here reduces the contribution both in the first and the second sequence, which reduces long-lasting effects.

## Discussion

This paper investigates the short-term and the long-lasting effects of various incentive mechanisms. In all treatments, these incentives are shown to be effective in increasing contributions when they are applied. Once they are removed, we observe long-lasting effects, and the end of the possibility to punish or reward does not lead to a direct dramatic drop in contributions. Rewards, in particular, appear to have much longer effects than punishments, and monetary incentives are more effective than non-monetary ones. While we observe significant differences throughout the period, the possibility of an absence of true long-lasting effects cannot be rejected, whereas we do not observe back-fired effects.

In our experiment, the end of the incentive mechanism does not seem to affect preferences or habits towards contribution. Our results also tend to confirm some of the empirical literature, especially in terms of energy and environmental conservation [1–15], which show the existence of long-lasting effects. The specific nature of our public good game without any framing may perhaps explain the convergence to the baseline at the end. There is no way to interest the subjects in an important societal question in our experiment that might perhaps have a long-term impact on their behavior.

It appears that having been heavily punished leads to some kind of revenge behavior in order to gain back what has been lost in the first periods. On the contrary, having been rewarded leads to some kind of delayed reciprocity, which explains why we have longer-lasting cooperation in the rewards treatments. This delayed reciprocity (or revenge behavior) could be compared to some learning or implementation of social norms at the neighborhood level that impacts the decision to continue to participate in the conservation of public good.

Our results also underline the necessity to look at the type of incentives used to design policies since non-monetary rewards have the same impact on contributions as monetary ones. In the particular case of public and social improvements, one reason to rely on programs based on non-monetary incentives concerns the sustainability of the funding possibilities and trust in institutions. In fact, monetary incentives can be costlier for institutions asking for individual contributions to a public good since they are difficult to quantify and often insufficient (i.e., not covering all the agents’ real costs). Furthermore, they can create some perverse effects (a positive contribution but a negative externality in another area), they are temporary and, finally, they can be rejected.

Our findings leave ample room for further research and new experiments. It seems particularly interesting to look at the dynamics of the incentives’ effects. Our results show that to sustain cooperation, it is important to maintain incentives with the question of periodicity that could be increasingly extended. The lessons from this experiment reinforce the idea that some long-lasting effects can be expected in a rewarding context. However, it is important to point out the rather short time of a specific laboratory experiment that lasted a little more than one hour. It would also be interesting to look at much longer-term effects and see if there are long-lasting effects when considering days, weeks or even months.

## Supporting information

S1 File Longlasting_incentives_plos_instructions. (DOCX)
