# Supplementary material for: Short- & long-term effects of monetary and non-monetary incentives to cooperate in public good games: An experiment
Source: PLoS One. 2020 Jan 17;15(1):e0227360. doi: 10.1371/journal.pone.0227360 (PMC6968839; doi:10.1371/journal.pone.0227360)
Supplement: S1 File — (DOCX) [file pone.0227360.s001.docx]

Instructions – MP translated

Thank you for participating in this experiment on decision making. In this experiment, your earnings depend on both your decisions and those of the other participants. We ask you to read these instructions carefully. They should allow you to understand the experiment. All your decisions are anonymous. You will never enter your name on the computer. Please indicate your choice on the computer at which you are sitting.

From now on, we ask you not to talk. If you have a question, please raise your hand and an experimenter will talk to you in private. It is forbidden to communicate with another participant during the experiment. If you violate this rule, you will be disqualified from the experiment and from any potential payment.

The 20 participants in the experiment are divided into groups of four, so that you are in a group with three other participants. You cannot know the identity of the other members of your group nor can the other members know your identity. You do not know the identity of the members of the other groups either. Your group will remain the same throughout the experiment. Your earnings will depend on your decisions and the decisions of other members of your group.

This experiment includes 30 successive periods divided into two sequences of 15 independent periods. In each period, you will earn gains calculated in tokens. At the end of the experiment, your total earnings in tokens accumulated over the 30 periods will be converted at the following rate:

30 tokens = €1

Gains in euros you have made ​​will then be paid in cash.

-------------------------------------------------------------------

**PERIODS 1-15**

Each of the first 15 periods is divided into two stages.

Stage 1

At the beginning of each period, you will receive 20 tokens. These 20 tokens constitute your initial endowment for this period. You must decide how to use this endowment. More precisely, you must decide how many tokens you want to invest in a common project with the group to which you belong and how many tokens you want to keep for yourself.

Specifically, at the beginning of each period, you decide the number of tokens between 0 and 20 that you want to invest in the common project. Choosing your investment in the project automatically determines the number of tokens you keep for yourself (20 minus your investment fees). For example, if you decide to invest 15 tokens in the project, you keep 5 tokens for yourself.

After each member of your group has made its investment choices, you are informed of the total amount invested in the project (i.e., your contribution and that of the others). You are also informed of your earnings for that period.

Your earnings for this period are the sum of two amounts:

1. The number of tokens you have not invested in the joint project and that you have kept for youself.

2. The income obtained through your investment in the joint project.

The investment in the joint project entitles you to an income. The income of the joint project is 40% of the total contributions to the project of the four members of the group (including your contribution).

Your gain for the period = (20 - your invested amount) + 0.4 * (the total of the invested amounts)

The income from the project is calculated in the same way for all members of your group, each group member thus receiving the same project income.

For example, if the total amount invested by the four members of the group is 60 tokens, each group member receives an income of 0.4 * 60 = 24 tokens. If the total investment is 9 tokens, each group member receives an income of 0.4 * 3.6 = 9 tokens from the project.

All the tokens that you do not invest in the joint project are for you. On the other hand, each token you spend for the joint project increases the total contribution of 1 token and therefore increases your income from the proposed 0.4 * 1 = 0.4 token. The income of other group members is also increased by 0.4 tokens in this case. Your investment in the joint project thus increases the income of other group members. Similarly, any investment in the joint project by another member of the group increases your own income and that of other group members.

- For example, if all group members keep their initial endowment of 20 tokens and do not contribute to the joint project, each group member keeps the 20 tokens that he or she was initially allotted and receives nothing from the project. The total gain for each member is 20 tokens.
- If all group members invest their entire initial allocation of 20 tokens in the project, the sum of the contributions is 80 tokens. Each group member will therefore receive an income of 32 tokens of the project and keep 0 tokens. The total gain for each member in this case is 32 tokens.

Stage 2

At the beginning of the second stage, you are informed of the level of individual contributions that each member of your group has made for the project. You then have the possibility to express your disapproval of each member by distributing points. You can distribute a large number of points to members of your group if you disagree with their decision to invest in the first step. You can give from 0 to a maximum of 10 points: 10 points if you strongly disagree with the decision and 0 points if you do not disapprove of the decision.

The other members of your group you can also distribute points if they wish. You must therefore decide for each member of your group, after learning of their contribution to the joint project, how many disapproval points you want to give. If you do not want to show your disapproval of a member, you can assign 0 disapproval points.

Remember that the same three participants and yourself form the group until the end of the experiment. However, you will not have the opportunity to identify each member individually. During each period, the investment in the joint project will be presented in ascending order without indication of the link between the investment and the group member who has made it.

Each point you give to another member of your group has a cost for you. Each point you give reduces your earnings from Stage 1 by 0.25 tokens.

• If you distribute 2 points to a member of your group, his/her earnings are reduced by 2 tokens and yours are reduced of 0.25 * 2 = 0.5 tokens. If you give 8 extra points to another member of your group, his/her earnings are reduced by 8 tokens but your earnings are reduced in total (2 + 8) * 0.25 = 2.5 tokens.

• If you give 0 points to a member of your group, neither his gains nor yours are affected.

The total amount of your earnings at the end of the period is calculated as follows:

| Gain for the period = earnings from Stage 1 - the sum of points received from other members - 0.25 * points given to the other members |
| --- |

Your earnings for the period can be negative if your earnings from Stage 1 are not sufficient to offset the points received and the costs of points distributed to other members.

Once all participants have made ​​their choice, your earnings for the period will be announced and another period will begin.

-------------------------------------------------------------------

**PERIODS 16-30**

During the periods from 16 to 30, you have to make the same decisions as in Stage 1 of periods 1 to 15. However, you will not have the opportunity to distribute points of disapproval to the members of your group. The other members of your group will no longer distribute disapproval points either. Therefore, neither you nor any member of your group can show its disapproval.

Your unique decision in each period will be to decide how much of your initial endowment of 20 tokens you want to invest in the joint project. The yield of the joint project is identical to the first 15 periods

Your earnings will be calculated in the same way as in Stage 1 of the first 15 periods:

Your gain for the period = (20 - your contribution) + 0.4 * (the total contributions)

Instructions - NP (translated)

Thank you for participating in this experiment on decision making. In this experiment, your earnings depend on both your decisions and those of the other participants. We ask you to read these instructions carefully. They should allow you to understand the experiment. All your decisions are anonymous. You will never enter your name on the computer. Please indicate your choice on the computer at which you are sitting.

From now on, we ask you not to talk. If you have a question, please raise your hand and an experimenter will talk to you in private. It is forbidden to communicate with another participant during the experiment. If you violate this rule, you will be disqualified from the experiment and from any potential payment.

The 20 participants in the experiment are divided into groups of four, so that you are in a group with three other participants. You cannot know the identity of the other members of your group nor can the other members know your identity. You do not know the identity of the members of the other groups either. Your group will remain the same throughout the experiment. Your earnings will depend on your decisions and the decisions of other members of your group.

This experiment includes 30 successive periods divided into two sequences of 15 independent periods. In each period, you will earn gains calculated in tokens. At the end of the experiment, your total earnings in tokens accumulated over the 30 periods will be converted at the following rate:

30 tokens = €1

Gains in euros you have made ​​will then be paid in cash.

-------------------------------------------------------------------

**PERIODS 1-15**

Each of the first 15 periods is divided into two stages.

Stage 1

At the beginning of each period, you will receive 20 tokens. These 20 tokens constitute your initial endowment for this period. You must decide how to use this endowment. More precisely, you must decide how many tokens you want to invest in a common project with the group to which you belong and how many tokens you want to keep for yourself.

Specifically, at the beginning of each period, you decide the number of tokens between 0 and 20 that you want to invest in the common project. Choosing your investment in the project automatically determines the number of tokens you keep for yourself (20 minus your investment fees). For example, if you decide to invest 15 tokens in the project, you keep 5 tokens for yourself.

After each member of your group has made its investment choices, you are informed of the total amount invested in the project (i.e., your contribution and that of the others). You are also informed of your earnings for that period.

Your earnings for this period are the sum of two amounts:

1. The number of tokens you have not invested in the joint project and that you have kept for youself.

2. The income obtained through your investment in the joint project.

The investment in the joint project entitles you to an income. The income of the joint project is 40% of the total contributions to the project of the four members of the group (including your contribution).

Your gain for the period = (20 - your invested amount) + 0.4 * (the total of the invested amounts)

The income from the project is calculated in the same way for all members of your group, each group member thus receiving the same project income.

For example, if the total amount invested by the four members of the group is 60 tokens, each group member receives an income of 0.4 * 60 = 24 tokens. If the total investment is 9 tokens, each group member receives an income of 0.4 * 3.6 = 9 tokens from the project.

All the tokens that you do not invest in the joint project are for you. On the other hand, each token you spend for the joint project increases the total contribution of 1 token and therefore increases your income from the proposed 0.4 * 1 = 0.4 token. The income of other group members is also increased by 0.4 tokens in this case. Your investment in the joint project thus increases the income of other group members. Similarly, any investment in the joint project by another member of the group increases your own income and that of other group members.

- For example, if all group members keep their initial endowment of 20 tokens and do not contribute to the joint project, each group member keeps the 20 tokens that he or she was initially allotted and receives nothing from the project. The total gain for each member is 20 tokens.
- If all group members invest their entire initial allocation of 20 tokens in the project, the sum of the contributions is 80 tokens. Each group member will therefore receive an income of 32 tokens of the project and keep 0 tokens. The total gain for each member in this case is 32 tokens.

Stage 2

At the beginning of the second stage, you are informed of the level of individual contributions that each member of your group has made for the project. You then have the possibility to express your disapproval of each member by distributing points. You can distribute a large number of points to members of your group if you disagree with their decision to invest in the first step. You can give from 0 to a maximum of 10 points: 10 points if you strongly disagree with the decision and 0 points if you do not disapprove of the decision.

The other members of your group you can also distribute points if they wish. You must therefore decide for each member of your group, after learning of their contribution to the joint project, how many disapproval points you want to give. If you do not want to show your disapproval of a member, you can assign 0 disapproval points.

Remember that the same three participants and yourself form the group until the end of the experiment. However, you will not have the opportunity to identify each member individually. During each period, the investment in the joint project will be presented in ascending order without indication of the link between the investment and the group member who has made it.

Remember that the same 3 participants and you form the group until the end of the experiment. However you will not have the opportunity to identify each member individually. During each period, the investment in the joint project will be presented in ascending order without indication of the link between investment and the group member who has achieved it.

Once all participants have made ​​their choice, your earnings for the period will be announced and another period will begin.

-------------------------------------------------------------------

**PERIODS 16-30**

During the periods from 16 to 30, you have to make the same decisions as in Stage 1 of periods 1 to 15. However, you will not have the opportunity to distribute points of disapproval to the members of your group. The other members of your group will no longer distribute disapproval points either. Therefore, neither you nor any member of your group can show its disapproval.

Your unique decision in each period will be to decide how much of your initial endowment of 20 tokens you want to invest in the joint project. The yield of the joint project is identical to the first 15 periods

Your earnings will be calculated in the same way as in Stage 1 of the first 15 periods:

Your gain for the period = (20 - your contribution) + 0.4 * (the total contributions)

Instructions – MP French

Nous vous remercions de participer à cette expérience sur la prise de décision. Dans cette expérience, vos gains dépendent **de vos décisions et de celles d’autres participants**. Nous vous demandons donc de lire attentivement ces instructions, elles doivent vous permettre de bien comprendre l’expérience. Toutes vos décisions sont **anonymes**. Vous n’entrerez jamais votre nom sur l’ordinateur. Vous indiquerez vos choix à l’ordinateur devant lequel vous êtes assis(e).

A partir de maintenant nous vous demandons de ne plus parler. Si vous avez une question levez la main et un expérimentateur viendra vous répondre en privé. Il est formellement interdit de communiquer avec un autre participant pendant l’expérience. Si vous ne respectez pas cette règle vous serez exclu de l’expérience et de tout paiement éventuel.

Les 20 participants à cette expérience sont répartis en groupe de 4. Vous faites donc partie d’un groupe avec trois autres participants. Vous ne pouvez pas connaitre l’identité des autres membres de votre groupe. De même qu’aucun membre de votre groupe ne peut connaitre votre identité. Vous ne connaissez pas non plus la constitution des autres groupes. Votre groupe restera identique tout le long de l'expérience. Vos gains dépendront de vos décisions et des décisions des autres membres de votre groupe.

Cette expérience comporte 30 périodes successives divisées en deux parties distinctes de 15 périodes indépendantes. Dans chaque période, vous pourrez accumulez des gains calculés en jetons. A la fin de l’expérience vos gains totaux en jetons accumulés au cours des 30 périodes seront convertis en euros au taux suivant :

30 jetons = 1 €

Les gains en euros que vous aurez réalisés vous seront alors versés en liquide.

-------------------------------------------------------------------

**PERIODES 1-15**

Chacune des 15 premières périodes comporte deux étapes.

Etape 1

Au début de chaque période vous recevez 20 jetons. Ces 20 jetons constituent votre dotation initiale pour cette période. Vous devez décider comment utiliser cette dotation. Plus précisément, vous devez décider combien de jetons vous voulez investir dans un projet commun au groupe dont vous êtes membre et combien de jetons vous voulez garder pour vous.

Concrètement, au début de chaque période vous décidez du nombre de jetons, entre 0 et 20 que vous voulez investir dans le projet commun. Le choix de votre investissement dans le projet détermine automatiquement le nombre de jetons que vous gardez pour vous (20 jetons moins votre investissement). *Par exemple si vous décidez d’investir 15 jetons dans le projet, vous gardez 5 jetons pour vous.*

Après que chaque membre de votre groupe a fait son choix d’investissement, vous êtes informé du montant total investi dans le projet (c’est-à-dire votre contribution plus celle des autres). Vous êtes également informé de vos gains pour cette période.

Vos gains pour cette période sont la somme de deux montants :

1. Le nombre de jetons que vous n’avez pas investis dans le projet commun et que vous avez gardés pour vous.
2. Le revenu obtenu via votre investissement dans le projet commun.

L’investissement dans le projet commun vous donne droit à un revenu. Ce revenu du projet commun correspond à 40% des contributions totales au projet des 4 membres du groupe (y compris votre contribution).

| Votre gain pour la période = (20 – le montant investi) + 0.4 * (le total des montants investis) |
| --- |

Le revenu provenant du projet est calculé de la même manière pour tous les membres de votre groupe, chaque membre du groupe reçoit donc le même revenu du projet.

- *Par exemple, si le total des montants investis par les 4 membres du groupe est 60 jetons, chaque membre du groupe reçoit un revenu de 0.4*60=24 jetons. Si l’investissement total est 9 jetons, chaque membre du groupe reçoit un revenu de 0.4*9=3.6 jetons provenant du projet.*

Tous les jetons que vous n’investissez pas dans le projet commun sont donc pour vous. Par contre, chaque jeton que vous dépensez pour le projet commun augmente la contribution totale de 1 jeton et augmente donc votre revenu provenant du projet de 0.4*1=0.4 jeton. Le revenu des autres membres du groupe est également augmenté de 0.4 jeton dans ce cas. Votre investissement dans le projet commun augmente donc le revenu des autres membres du groupe. De façon similaire, tout investissement au projet commun par un autre membre du groupe augmente votre propre revenu ainsi que celui des autres membres du groupe.

- *Par exemple, si tous les membres du groupe gardent leur dotation initiale de 20 jetons et ne contribuent pas au projet commun, chaque membre du groupe reçoit les 20 jetons qu’il ou elle a gardé et ne reçoit rien provenant du projet. Le gain total pour chaque membre est de 20 jetons.*
- *Si tous les membres du groupe investissent la totalité de leur dotation initiale de 20 jetons dans le projet, la somme des contributions est donc 80 jetons. Chaque membre du groupe recevra donc un revenu de 32 jetons du projet et 0 jeton gardé. Le gain total pour chaque membre est 32 jetons.*

Etape 2

Au début de la seconde étape, vous êtes informé du niveau des contributions individuelles que chaque membre de votre groupe a fait pour le projet. Vous avez alors la possibilité de réduire le revenu de chaque membre du groupe en distribuant des points. Vous pouvez distribuer un grand nombre de points à un membre de votre groupe si vous désapprouvez sa décision d’investissement dans la première étape. Vous pouvez donner entre 0 et 10 points maximum: 10 points si vous désapprouvez fortement sa décision à 0 point si vous ne désapprouvez pas sa décision. Chaque point de désapprobation diminue de 1 jeton le revenu obtenu lors de la première étape.

Les autres membres de votre groupe peuvent également diminuer votre gain de l’étape 1 s’ils le désirent. Vous devez donc décider pour chaque membre de votre groupe, après avoir pris connaissance de leur investissement dans le projet commun, combien de points de désapprobation vous voulez lui donner. Si vous ne voulez pas changer le gain d’un membre, vous pouvez lui assigner 0 point de désapprobation.

N’oubliez pas que les mêmes 3 participants et vous-même formeront le groupe jusqu’à la fin de l’expérience. Cependant vous n’aurez pas la possibilité d’identifier chaque membre individuellement. Lors de chaque période, les investissements réalisés dans le projet commun vous seront présentés en ordre croissant sans indication du lien entre un investissement et le membre du groupe qui l’a réalisé.

Chaque point que vous donnez à un autre membre de votre groupe a un coût pour vous. Chaque point que vous donnez réduit vos gains de l’étape 1 de 0.25 jetons.

- *Si vous distribuez 2 points à un membre de votre groupe. Ses gains sont réduit de 2 jetons et les vôtres sont réduit de 0.25 * 2 = 0.5 jetons. Si vous donnez 8 jetons supplémentaires à un autre membre de votre groupe, ses gains sont diminués de 8 jetons mais vos gains sont diminués au total de (2+8) * 0.25 = 2.5 jetons.*
- *Si vous donnez 0 points à un membre de votre groupe, ni ses gains ni les vôtres ne sont affectés.*

Le montant total de vos gains à la fin de la période est calculé comme ceci :

| Gain de la période = gains de l’étape 1  – somme des points reçus des autres membres  – 0.25 * les points distribués aux autres membres |
| --- |

Vos gains pour la période peuvent donc être négatifs si vos gains de l’étape 1 ne sont pas suffisants pour compenser les points reçus et les coûts des points distribués aux autres membres.

Une fois que tous les participants auront fait leur choix, vos gains pour la période vous seront communiqués et une autre période commencera.

-------------------------------------------------------------------

**PERIODES 16-30**

Au cours des périodes 16 à 30, vous aurez à prendre les mêmes décisions que lors de l’étape 1 des périodes 1 à 15. **Par contre vous n’aurez plus l’occasion de distribuer des points de désapprobation aux membres de votre groupe.** Les autres membres de votre groupe ne pourront plus distribuer de points non plus. Donc vous ne pourrez plus réduire les gains des membres de votre groupe, de même qu’aucun membre de votre groupe ne pourra diminuer vos gains.

Votre seule décision, au cours de chaque période, consistera à décider combien de vos 20 jetons de dotation initiale vous voulez investir dans le projet commun. Le rendement du projet commun est identique aux 15 premières périodes.

Vos gains seront calculés de la même façon que lors de l’étape 1 dans les 15 premières périodes :

| Votre gain pour la période = (20 - votre contribution) + 0.4 * (le total des contributions) |
| --- |

Instructions – NB (French)

Nous vous remercions de participer à cette expérience sur la prise de décision. Dans cette expérience, vos gains dépendent **de vos décisions et de celles d’autres participants**. Nous vous demandons donc de lire attentivement ces instructions, elles doivent vous permettre de bien comprendre l’expérience. Toutes vos décisions sont **anonymes**. Vous n’entrerez jamais votre nom sur l’ordinateur. Vous indiquerez vos choix à l’ordinateur devant lequel vous êtes assis(e).

A partir de maintenant nous vous demandons de ne plus parler. Si vous avez une question levez la main et un expérimentateur viendra vous répondre en privé. Il est formellement interdit de communiquer avec un autre participant pendant l’expérience. Si vous ne respectez pas cette règle vous serez exclu de l’expérience et de tout paiement éventuel.

Les 20 participants à cette expérience sont répartis en groupe de 4. Vous faites donc partie d’un groupe avec trois autres participants. Vous ne pouvez pas connaitre l’identité des autres membres de votre groupe. De même qu’aucun membre de votre groupe ne peut connaitre votre identité. Vous ne connaissez pas non plus la constitution des autres groupes. Votre groupe restera identique tout le long de l'expérience. Vos gains dépendront de vos décisions et des décisions des autres membres de votre groupe.

Cette expérience comporte 30 périodes successives divisées en deux parties distinctes de 15 périodes indépendantes. Dans chaque période, vous pourrez accumulez des gains calculés en jetons. A la fin de l’expérience vos gains totaux en jetons accumulés au cours des 30 périodes seront convertis en euros au taux suivant :

30 jetons = 1 €

Les gains en euros que vous aurez réalisés vous seront alors versés en liquide.

-------------------------------------------------------------------

**PERIODES 1-15**

Chacune des 15 premières périodes comporte deux étapes.

Etape 1

Au début de chaque période vous recevez 20 jetons. Ces 20 jetons constituent votre dotation initiale pour cette période. Vous devez décider comment utiliser cette dotation. Plus précisément, vous devez décider combien de jetons vous voulez investir dans un projet commun au groupe dont vous êtes membre et combien de jetons vous voulez garder pour vous.

Concrètement, au début de chaque période vous décidez du nombre de jetons, entre 0 et 20 que vous voulez investir dans le projet commun. Le choix de votre investissement dans le projet détermine automatiquement le nombre de jetons que vous gardez pour vous (20 jetons moins votre investissement). *Par exemple si vous décidez d’investir 15 jetons dans le projet, vous gardez 5 jetons pour vous.*

Après que chaque membre de votre groupe a fait son choix d’investissement, vous êtes informé du montant total investi dans le projet (c’est-à-dire votre contribution plus celle des autres). Vous êtes également informé de vos gains pour cette période.

Vos gains pour cette période sont la somme de deux montants :

1. Le nombre de jetons que vous n’avez pas investis dans le projet commun et que vous avez gardés pour vous.
2. Le revenu obtenu via votre investissement dans le projet commun.

L’investissement dans le projet commun vous donne droit à un revenu. Ce revenu du projet commun correspond à 40% des contributions totales au projet des 4 membres du groupe (y compris votre contribution).

| Votre gain pour la période = (20 – le montant investi) + 0.4 * (le total des montants investis) |
| --- |

Le revenu provenant du projet est calculé de la même manière pour tous les membres de votre groupe, chaque membre du groupe reçoit donc le même revenu du projet.

- *Par exemple, si le total des montants investis par les 4 membres du groupe est 60 jetons, chaque membre du groupe reçoit un revenu de 0.4*60=24 jetons. Si l’investissement total est 9 jetons, chaque membre du groupe reçoit un revenu de 0.4*9=3.6 jetons provenant du projet.*

Tous les jetons que vous n’investissez pas dans le projet commun sont donc pour vous. Par contre, chaque jeton que vous dépensez pour le projet commun augmente la contribution totale de 1 jeton et augmente donc votre revenu provenant du projet de 0.4*1=0.4 jeton. Le revenu des autres membres du groupe est également augmenté de 0.4 jeton dans ce cas. Votre investissement dans le projet commun augmente donc le revenu des autres membres du groupe. De façon similaire, tout investissement au projet commun par un autre membre du groupe augmente votre propre revenu ainsi que celui des autres membres du groupe.

- *Par exemple, si tous les membres du groupe gardent leur dotation initiale de 20 jetons et ne contribuent pas au projet commun, chaque membre du groupe reçoit les 20 jetons qu’il ou elle a gardé et ne reçoit rien provenant du projet. Le gain total pour chaque membre est de 20 jetons.*
- *Si tous les membres du groupe investissent la totalité de leur dotation initiale de 20 jetons dans le projet, la somme des contributions est donc 80 jetons. Chaque membre du groupe recevra donc un revenu de 32 jetons du projet et 0 jeton gardé. Le gain total pour chaque membre est 32 jetons.*

Etape 2

Au début de la seconde étape, vous êtes informé du niveau des contributions individuelles que chaque membre de votre groupe a fait pour le projet. Vous avez alors la possibilité d’exprimer votre désapprobation à l’encontre de chaque membre du groupe en distribuant des points. Vous pouvez distribuer un grand nombre de points à un membre de votre groupe si vous désapprouvez sa décision d’investissement dans la première étape. Vous pouvez donner entre 0 et 10 points maximum: 10 points si vous désapprouvez fortement sa décision à 0 point si vous ne désapprouvez pas sa décision.

Les autres membres de votre groupe peuvent également vous distribuer des points 1 s’ils le désirent. Vous devez donc décider pour chaque membre de votre groupe, après avoir pris connaissance de leur contribution au projet commun, combien de points de désapprobation vous voulez lui donner. Si vous ne voulez pas montre votre désapprobation à l’encontre d’un membre, vous pouvez lui assigner 0 point de désapprobation.

N’oubliez pas que les mêmes 3 participants et vous-même formeront le groupe jusqu’à la fin de l’expérience. Cependant vous n’aurez pas la possibilité d’identifier chaque membre individuellement. Lors de chaque période, les investissements réalisés dans le projet commun vous seront présentés en ordre croissant sans indication du lien entre un investissement et le membre du groupe qui l’a réalisé.

Une fois que tous les participants auront fait leur choix, vos gains pour la période vous seront communiqués et une autre période commencera.

-------------------------------------------------------------------

**PERIODES 16-30**

Au cours des périodes 16 à 30, vous aurez à prendre les mêmes décisions que lors de l’étape 1 des périodes 1 à 15. **Par contre vous n’aurez plus l’occasion de distribuer des points de désapprobation aux membres de votre groupe**. Les autres membres de votre groupe ne pourront plus distribuer de points non plus. Donc vous ne pourrez plus montrer votre désapprobation, de même qu’aucun membre de votre groupe ne pourra montrer la sienne.

Votre seule décision, au cours de chaque période, consistera à décider combien de vos 20 jetons de dotation initiale vous voulez investir dans le projet commun. Le rendement du projet commun est identique aux 15 premières périodes.

Vos gains seront calculés de la même façon que lors de l’étape 1 dans les 15 premières périodes :

| Votre gain pour la période = (20 - votre contribution) + 0.4 * (le total des contributions) |
| --- |

Instructions – MR (French)

Nous vous remercions de participer à cette expérience sur la prise de décision. Dans cette expérience, vos gains dépendent **de vos décisions et de celles d’autres participants**. Nous vous demandons donc de lire attentivement ces instructions, elles doivent vous permettre de bien comprendre l’expérience. Toutes vos décisions sont **anonymes**. Vous n’entrerez jamais votre nom sur l’ordinateur. Vous indiquerez vos choix à l’ordinateur devant lequel vous êtes assis(e).

A partir de maintenant nous vous demandons de ne plus parler. Si vous avez une question levez la main et un expérimentateur viendra vous répondre en privé. Il est formellement interdit de communiquer avec un autre participant pendant l’expérience. Si vous ne respectez pas cette règle vous serez exclu de l’expérience et de tout paiement éventuel.

Les 20 participants à cette expérience sont répartis en groupe de 4. Vous faites donc partie d’un groupe avec trois autres participants. Vous ne pouvez pas connaitre l’identité des autres membres de votre groupe. De même qu’aucun membre de votre groupe ne peut connaitre votre identité. Vous ne connaissez pas non plus la constitution des autres groupes. Votre groupe restera identique tout le long de l'expérience. Vos gains dépendront de vos décisions et des décisions des autres membres de votre groupe.

Cette expérience comporte 30 périodes successives divisées en deux parties distinctes de 15 périodes indépendantes. Dans chaque période, vous pourrez accumulez des gains calculés en jetons. A la fin de l’expérience vos gains totaux en jetons accumulés au cours des 30 périodes seront convertis en euros au taux suivant :

30 jetons = 1 €

Les gains en euros que vous aurez réalisés vous seront alors versés en liquide.

-------------------------------------------------------------------

**PERIODES 1-15**

Chacune des 15 premières périodes comporte deux étapes.

Etape 1

Au début de chaque période vous recevez 20 jetons. Ces 20 jetons constituent votre dotation initiale pour cette période. Vous devez décider comment utiliser cette dotation. Plus précisément, vous devez décider combien de jetons vous voulez investir dans un projet commun au groupe dont vous êtes membre et combien de jetons vous voulez garder pour vous.

Concrètement au début de chaque période vous décidez du nombre de jetons, entre 0 et 20 que vous voulez investir dans le projet commun. Le choix de votre investissement dans le projet détermine automatiquement le nombre de jetons que vous gardez pour vous (20 jetons moins votre investissement). Par exemple si vous décidez d’investir 15 jetons dans le projet, vous gardez 5 jetons pour vous.

Après que chaque membre de votre groupe a fait son choix d’investissement, vous êtes informé du montant total investi dans le projet (c’est-à-dire votre contribution plus celle des autres). Vous êtes également informé de vos gains pour cette période.

Vos gains pour cette période sont la somme de deux montants :

1. Le nombre jetons que vous n’avez pas investi dans le projet commun et que vous avez gardé pour vous.
2. Le revenu obtenu via votre investissement dans le projet commun.

L’investissement dans le projet commun vous donne droit à un revenu. Ce revenu du projet commun correspond à 40% des contributions totales au projet des 4 membres du groupe (y compris votre contribution).

| Votre gain pour la période = (20 – le montant investi) + 0.4 * (le total des montants investis) |
| --- |

Le revenu provenant du projet est calculé de la même manière pour tous les membres de votre groupe, chaque membre du groupe reçoit donc le même revenu du projet.

- *Par exemple, si le total des montants investis par les 4 membres du groupe est 60 jetons, chaque membre du groupe reçoit un revenu de 0.4*60=24 jetons. Si l’investissement total est 9 jetons, chaque membre du groupe reçoit un revenu de 0.4*9=3.6 jetons provenant du projet.*

Tous les jetons que vous n’investissez pas dans le projet commun sont donc pour vous. Par contre, chaque jeton que vous dépensez pour le projet commun augmente la contribution totale de 1 jeton et augmente donc votre revenu provenant du projet de 0.4*1=0.4 jeton. Le revenu des autres membres du groupe est également augmenté de 0.4 jeton dans ce cas. Votre investissement dans le projet commun augmente donc le revenu des autres membres du groupe. De façon similaire, tout investissement au projet commun par un autre membre du groupe augmente votre propre revenu ainsi que celui des autres membres du groupe.

- *Par exemple, si tous les membres du groupe gardent leur dotation initiale de 20 jetons et ne contribuent pas au projet commun, chaque membre du groupe reçoit les 20 jetons qu’il ou elle a gardé et ne reçoit rien provenant du projet. Le gain total pour chaque membre est de 20 jetons.*
- *Si tous les membres du groupe investissent la totalité de leur dotation initiale de 20 jetons dans le projet, la somme des contributions est donc 80 jetons. Chaque membre du groupe recevra donc un revenu de 32 jetons du projet et 0 jeton gardé. Le gain total pour chaque membre est 32 jetons.*

Etape 2

Au début de la seconde étape, vous êtes informé du niveau des contributions individuelles que chaque membre de votre groupe a fait pour le projet. Vous avez alors la possibilité de récompenser chaque membre du groupe en distribuant des points, et donc augmenter leur revenu. Vous pouvez distribuer un grand nombre de points à un membre de votre groupe si vous approuvez sa décision d’investissement dans la première étape. Vous pouvez donner entre 0 et 10 points maximum: 10 points si vous approuvez fortement sa décision à 0 point si vous n’approuvez pas sa décision. Chaque point de récompense augmente de 1 jeton le revenu obtenu lors de la première étape.

Les autres membres de votre groupe peuvent également augmenter votre gain de l’étape 1 s’ils le désirent. Vous devez donc décider pour chaque membre de votre groupe, après avoir pris connaissance de leur contribution au projet commun, combien de points de récompense vous voulez lui donner. Si vous ne voulez pas changer le gain d’un membre, vous pouvez lui assigner 0 point de récompense.

N’oubliez pas que les mêmes 3 participants et vous-même formeront le groupe jusqu’à la fin de l’expérience. Cependant vous n’aurez pas la possibilité d’identifier chaque membre individuellement. Lors de chaque période, les investissements réalisés dans le projet commun vous seront présentés en ordre croissant sans indication du lien entre un investissement et le membre du groupe qui l’a réalisé.

Chaque point que vous donnez à un autre membre de votre groupe a un coût pour vous. Chaque point que vous donnez réduit vos gains de l’étape 1 de 0.25 jetons.

- *Si vous distribuez 2 points à un membre de votre groupe. Ses gains sont augmentés de 2 jetons et les vôtres sont réduit de 0.25 * 2 = 0.5 jetons. Si vous donnez 8 points supplémentaires à un autre membre de votre groupe, ses gains sont augmentés de 8 jetons mais vos gains sont diminués au total de (2+8) * 0.25 = 2.5 jetons.*
- *Si vous donnez 0 points à un membre de votre groupe, ni ses gains ni les vôtres ne sont affectés.*

Le montant total de vos gains à la fin de la période est calculé comme ceci :

| Gain de la période = gains de l’étape 1  + somme des points reçus des autres membres  – 0.25 * les points distribués aux autres membres |
| --- |

Vos gains pour la période peuvent donc être négatifs si vos gains de l’étape 1 ne sont pas suffisants pour compenser les coûts des points distribués aux autres membres.

Une fois que tous les participants auront fait leur choix, vos gains pour la période vous seront communiqués et une autre période commencera.

-------------------------------------------------------------------

**PERIODES 16-30**

Au cours des périodes 16 à 30, vous aurez à prendre les mêmes décisions que lors de l’étape 1 des périodes 1 à 15. **Par contre vous n’aurez plus l’occasion de distribuer des points de récompense aux membres de votre groupe**. Les autres membres de votre groupe ne pourront plus distribuer de points non plus. Donc vous ne pourrez plus récompenser les membres de votre groupe, de même qu’aucun membre de votre groupe ne pourra vous récompenser.

Votre seule décision, au cours de chaque période, consistera à décider combien de vos 20 jetons de dotation initiale vous voulez investir dans le projet commun. Le rendement du projet commun est identique aux 15 premières périodes.

Vos gains seront calculés de la même façon que lors de l’étape 1 dans les 15 premières périodes :

| Votre gain pour la période = (20 - votre contribution) + 0.4 * (le total des contributions) |
| --- |

Instructions – NR (French)

Nous vous remercions de participer à cette expérience sur la prise de décision. Dans cette expérience, vos gains dépendent **de vos décisions et de celles d’autres participants**. Nous vous demandons donc de lire attentivement ces instructions, elles doivent vous permettre de bien comprendre l’expérience. Toutes vos décisions sont **anonymes**. Vous n’entrerez jamais votre nom sur l’ordinateur. Vous indiquerez vos choix à l’ordinateur devant lequel vous êtes assis(e).

A partir de maintenant nous vous demandons de ne plus parler. Si vous avez une question levez la main et un expérimentateur viendra vous répondre en privé. Il est formellement interdit de communiquer avec un autre participant pendant l’expérience. Si vous ne respectez pas cette règle vous serez exclu de l’expérience et de tout paiement éventuel.

Les 20 participants à cette expérience sont répartis en groupe de 4. Vous faites donc partie d’un groupe avec trois autres participants. Vous ne pouvez pas connaitre l’identité des autres membres de votre groupe. De même qu’aucun membre de votre groupe ne peut connaitre votre identité. Vous ne connaissez pas non plus la constitution des autres groupes. Votre groupe restera identique tout le long de l'expérience. Vos gains dépendront de vos décisions et des décisions des autres membres de votre groupe.

Cette expérience comporte des périodes successives divisées en deux parties distinctes de périodes indépendantes.. Dans chaque période, vous pourrez accumuler des gains calculés en jetons. A la fin de l’expérience vos gains totaux en jetons accumulés au cours des périodes seront convertis en euros au taux suivant :

30 jetons = 1 €

Les gains en euros que vous aurez réalisés vous seront alors versés en liquide.

-------------------------------------------------------------------

**PARTIE 1 : PERIODES 1-15**

Chacune des 15 premières périodes comporte deux étapes.

Etape 1

Au début de chaque période vous recevez 20 jetons. Ces 20 jetons constituent votre dotation initiale pour cette période. Vous devez décider comment utiliser cette dotation. Plus précisément, vous devez décider combien de jetons vous voulez investir dans un projet commun au groupe dont vous êtes membre et combien de jetons vous voulez garder pour vous.

Concrètement au début de chaque période vous décidez du nombre de jetons, entre 0 et 20 que vous voulez investir dans le projet commun. Le choix de votre investissement dans le projet détermine automatiquement le nombre de jetons que vous gardez pour vous (20 jetons moins votre investissement). Par exemple si vous décidez d’investir 15 jetons dans le projet, vous gardez 5 jetons pour vous.

Après que chaque membre de votre groupe a fait son choix d’investissement, vous êtes informé du montant total investi dans le projet (c’est-à-dire votre contribution plus celle des autres). Vous êtes également informé de vos gains pour cette période.

Vos gains pour cette période sont la somme de deux montants :

1. Le nombre jetons que vous n’avez pas investi dans le projet commun et que vous avez gardé pour vous.
2. Le revenu obtenu via votre investissement dans le projet commun.

L’investissement dans le projet commun vous donne droit à un revenu. Ce revenu du projet commun correspond à 40% des contributions totales au projet des 4 membres du groupe (y compris votre contribution).

| Votre gain pour la période = (20 – le montant investi) + 0.4 * (le total des montants investis) |
| --- |

Le revenu provenant du projet est calculé de la même manière pour tous les membres de votre groupe, chaque membre du groupe reçoit donc le même revenu du projet.

- *Par exemple, si le total des montants investis par les 4 membres du groupe est 60 jetons, chaque membre du groupe reçoit un revenu de 0.4*60=24 jetons. Si l’investissement total est 9 jetons, chaque membre du groupe reçoit un revenu de 0.4*9=3.6 jetons provenant du projet.*

Tous les jetons que vous n’investissez pas dans le projet commun sont donc pour vous. Par contre, chaque jeton que vous dépensez pour le projet commun augmente la contribution totale de 1 jeton et augmente donc votre revenu provenant du projet de 0.4*1=0.4 jeton. Le revenu des autres membres du groupe est également augmenté de 0.4 jeton dans ce cas. Votre investissement dans le projet commun augmente donc le revenu des autres membres du groupe. De façon similaire, tout investissement au projet commun par un autre membre du groupe augmente votre propre revenu ainsi que celui des autres membres du groupe.

- *Par exemple, si tous les membres du groupe gardent leur dotation initiale de 20 jetons et ne contribuent pas au projet commun, chaque membre du groupe reçoit les 20 jetons qu’il ou elle a gardé et ne reçoit rien provenant du projet. Le gain total pour chaque membre est de 20 jetons.*
- *Si tous les membres du groupe investissent la totalité de leur dotation initiale de 20 jetons dans le projet, la somme des contributions est donc 80 jetons. Chaque membre du groupe recevra donc un revenu de 32 jetons du projet et 0 jeton gardé. Le gain total pour chaque membre est 32 jetons.*

Etape 2

Au début de la seconde étape, vous êtes informé du niveau des contributions individuelles que chaque membre de votre groupe a fait pour le projet. Vous avez alors la possibilité d’exprimer votre approbation à l’encontre de chaque membre du groupe en distribuant des points de récompense. Vous pouvez distribuer un grand nombre de points à un membre de votre groupe si vous approuvez sa décision d’investissement dans la première étape. Vous pouvez donner entre 0 et 10 points maximum: 10 points si vous approuvez fortement sa décision à 0 point si vous n’approuvez pas sa décision.

Les autres membres de votre groupe peuvent également vous distribuer des points de récompense s’ils le désirent. Vous devez donc décider pour chaque membre de votre groupe, après avoir pris connaissance de leur contribution au projet commun, combien de points d’approbation vous voulez lui donner. Si vous ne voulez pas donner de points à un membre de votre groupe, vous pouvez lui assigner 0 point d’approbation.

N’oubliez pas que les mêmes 3 participants et vous-même formeront le groupe jusqu’à la fin de l’expérience. Cependant vous n’aurez pas la possibilité d’identifier chaque membre individuellement. Lors de chaque période, les investissements réalisés dans le projet commun vous seront présentés en ordre croissant sans indication du lien entre un investissement et le membre du groupe qui l’a réalisé.

Une fois que tous les participants auront fait leur choix, vos gains pour la période vous seront communiqués et une autre période commencera.

-------------------------------------------------------------------

**PERIODES 16-30**

Au cours des périodes 16 à 30, vous aurez à prendre les mêmes décisions que lors de l’étape 1 des périodes 1 à 15. **Par contre vous n’aurez plus l’occasion de distribuer des points de récompense aux membres de votre groupe**. Les autres membres de votre groupe ne pourront plus distribuer de points non plus. Donc vous ne pourrez plus récompenser les membres de votre groupe, de même qu’aucun membre de votre groupe ne pourra vous récompenser.

Votre seule décision, au cours de chaque période, consistera à décider combien de vos 20 jetons de dotation initiale vous voulez investir dans le projet commun. Le rendement du projet commun est identique aux 15 premières périodes.

Vos gains seront calculés de la même façon que lors de l’étape 1 dans les 15 premières périodes :

| Votre gain pour la période = (20 - votre contribution) + 0.4 * (le total des contributions) |
| --- |
